# Lead as a Possible Cause of Azoturia

**Published:** 1900-08

**Authors:** 


### SELECTION.

### LEAD AS A POSSIBLE CAUSE OF AZOTURIA.

Dr. A. W. Balch (*Journal of the Boston Society of Medical Sciences*, May), owing to the similarity between certain of the symptoms existing in azoturia and those commonly found in lead-poisoning, examined the urine and tissue-specimen taken from normal horses. In both urine and tissues lead was found in cases other than azoturia, but the amounts were much larger and the lead was much more widely distributed in the azoturia cases. The organs examined included brain, liver, spleen, kidney, and psoas muscle.

Dr. W. R. Brinckerhoff, supplementingDr. Balch's chemical investigations in the same journal, as a result of eleven autopsies on undoubted cases of azoturia, reaches the following conclusions.

1. The acute degenerations of the liver and kidney, the proliferation of epithelioid cells in the lymph-follicles of the spleen, and the focal necroses in the liver, are similar to lesions found in man during various of the acute infectious diseases. In the case of the human being such changes are generally regarded as manifestations of a toxæmia.

2. The pigmentation of the spleen indicates nothing more than a destruction of blood-corpuscles, and is common to a number of diseases in both man and animals.

3. The degeneration of the psoas muscle is quite analogous to the Zenker's degeneration seen in typhoid fever.

4. The chronic interstitial nephritis would indicate either that some toxic substance had acted for a considerable time, or that there had been previous attacks of an acute nature.

We have, then, the picture of a pathological process combining chronic and acute factors. This condition seems most reasonably explained by postulating the presence in the organism of a toxic substance, preferably one capable of causing both acute and chronic changes. *New York Medical Journal*, August 4, 1900.

---

ONE has but to take a cursory glance of *Bulletin No.* 66 of the West Virginia Agricultural Experiment Station to learn of the category of fruit-diseases and the remedies for their treatment and eradication. Successful fruit-culture is no longer a matter of luck, no more than successful breeding and rearing of animals. Eternal vigilance after the enemies of animate and inanimate nature is the watchword of success. Alike in fruit diseases as in animal diseases, " a stitch in time saves nine." These are lessons well learned, and the amount of valuable information on these lines furnished by our Experiment Station staff brings results worth thrice the cost of maintenance of these important centres of knowledge.

